# Waste slag benefits for correction of soil acidity

**DOI:** 10.1038/s41598-022-20528-6

**Published:** 2022-09-26

**Authors:** Viorica Ghisman, Alina Crina Muresan, Daniela Laura Buruiana, Elena Roxana Axente

**Affiliations:** 1 Interdisciplinary Research Centre in the Field of Eco-Nano Technology and Advance Mate-Rials CC-ITI, Faculty of Engineering, “Dunarea de Jos” University of Galati, 47 Domneasca, 800008 Galati, Romania; 2 Medicine and Pharmacy Faculty, “Dunarea de Jos” University of Galati, 47 Domneasca, 800008 Galati, Romania

**Keywords:** Ecology, Environmental sciences, Engineering, Materials science

## Abstract

The global trend is to find new materials with improved environment friendly. The sustainable development of 2030 AGENDA and Waste Management Legislation sustain the disposal of a large quantity of slag at landfill sites by causing environmental consequences which has drawn attention to the need for its more effective recycling. Heavy industries have been operating in the Galati area for over 30 years and an ecological education is necessary for an efficient management of waste slag. The agricultural land resources are an issue world-wide and through this investigative study we showed that the mixture of blast furnace slag and waste slag dumped in landfill can help remediation of the soil acidity and increasing the crop yield. The chemical, structural and morphological properties of three investigated different slag samples are evaluated for recycling in agriculture. Results indicated that the obtained mixture of the slag waste dumped in landfill and of granulated metallurgical slag shows its usage in saving the affected lands. Therefore, by elemental analysis determined by X-ray fluorescence analytical equipment, the optimum weight ratio for the composition of soil-slag mixture were achieved. The obtained mixture presents a balance between soil pH = 5.2 corresponding to a medium acid soil and slag pH = 12.5 which corresponds as strongly basic character which is beneficial in amelioration process of acidic soils for the improving of soil characteristics.

## Introduction

In the past, steel industries were designed to produce steel of a specific quality and quantity, without any monitorization in environmental protection, which led to the increase of higher volume of by-products (slag). The disposal of a large quantity of slag at landfill sites caused environmental consequences which has drawn the attention to the need for its more effective recycling^1^. Furthermore, the land filled with dumped slag has become an important source of pollution of environmental factors which for-wards adversely affects human health from the perspective of cardiovascular and respiratory health problems^2–4^.

The various waste slags that are emerged from steel plants are blast furnace slag (BFS) and Linz-Donawitz (LD) converter slag. The composition of these slags differs depending on the source of generation but consists of oxides such as silica, alumina, iron, lime, and magnesia^5^. For many years, due to the rapid growth of industrialization, the quantity of metallurgical slag deposited in slag storing yards, occupied farmland and silted rivers has reached to enormous amounts^6^.

Over the past decade, the increased number of environmental regulations corroborated with rising costs and decreasing capacity at landfills have forced the steel industries to minimize the waste disposal and to find ways to resource recovery and reuse of waste slags. Nowadays, the slags coming from blast furnace and steelmaking processes are considered marketable products used in cement, roadbed material, ground improvement material, civil engineering material, fertilizer and only a small percentage is processed as waste in landfills. During the production of steel, one ton of slag waste is generated per every ton of stainless steel produced^7^. One important contribution in saving of natural resources, CO_2_ emissions reduction and energy consumption reduction consists in sustainable use of slags to form a society founded on the recycling practice. The principal objective of the program HORIZON 2020 of the EU is to recover and recycle resources from metallurgical wastes and to support friendly ecological economy^8–10^.

Particularly, the largest Integrated Steel Plant of Galati, located in South-East Romania, is a leader in manufacturing metallurgical products, with a current production capacity of 3 million tons of steel per year as well as the ability to increase that output in the future. With a history from 1968 the Steel Plant of Galati, with a surface area of 1600 ha, when had a production for over 8 million tons of steel/year and 12,000 of employees. The Slag Dump of Steel Plant of Galati has a surface area of 110 ha and an occupied capacity of 70 million tons of slag. The Slag Dump was closed in 2009, but the waste slag was not recycled and currently has a height of 60 m.

The slag wastes dumped in landfills cannot be directed to material, raw material, or chemical recycling due to their high heterogeneity. Also, the authors^11^ evaluated the chemical and significant geotechnical parameters of slag collected from a metallurgical landfill as the alternative materials used in roads construction. In previous research we studied about the re-use of slag with application in industrial sector with material and environmental benefits^12^.

Agricultural acid soils (pH value less than 7) have low fertility, poor physical, chemical, and biological properties which seriously affect the crops yield^13^. Soil acidity can cause molybdenum, calcium, and magnesium deficiency in plants and de-creases phosphorous availability^14^. Because the land resources are limited, it is necessary to pay attention to the correction of soil acidity and to the increase of the crop yield. In Japan and Europe steel slag is used as soil amendment agent since it contains a variety of trace elements on its surface, such as Cr and V, which are not readily released in slag. Also, the presence of Ca^2+^ on the surface of steel slag produces stable potentially toxic element (PTE) ions, with the purpose to immobilize PTE ions from contaminated soil^15,16^. Wen et al.^17^ showed that applying steel slag onto PTE acidic mining soils effectively increases the soil pH and soil microbial abundance and immobilizes PTE ions which provide a desirable plant survival climate. Popescu et al.^18^ investigated three sources of ladle furnace (LD) slag from Romanian steel plants with characteristics regarding steel manufacturing and storage of LF slag which exploit them as environmentally friendly secondary resources to obtain materials for improving acidic soils. Anger et al.^19^ investigated the effects of ladle furnace slag presence on soil properties, and they concluded that the use of ladle furnace slag for acid soil remediation is effective with a positive influence on soil characteristics, leading to increased crop yield. Mihalache et al.^20^ concluded that the waste resulted from secondary refinery of the steel can be use with good results in agriculture, with positive influences on the maize crop and as amendment for correction acidic soil reaction, and the slag from the steel industry brings increases of calcium and micronutrients in soil content. Fan et al.^21^ concluded that the use of slag as a fertilizer can decrease Cr, Cu, Pb and Zn in acidic soils and indicated that the slag could be used in PTE pollution control for plants and the environment. The researchers^22^ investigated the feasibility of using BOF slag for degraded land remediation in two different ways: as liming material and as amendment nutrient source and they showed that as the slag content increases, the mobility of some basic nutrients such as Mg decreases, so an addition of more than 25% is not recommended. Dongfeng Ning et. al.^23^ carried out a pot experiments to assess the effect of application rates of steel slag on improving soil acidity and silicon-availability to demonstrate the effects of steel slags on the immobilization of heavy metals in contaminated soil, to test the impacts of slag application on rice productivity and metals accumulation and they concluded that steel slag was an effective amendment for soil acidity adjustment, plant silicon nutrition and stabilization of Cd in acidic soils.

From perspective of waste directive 2008/98/EC (EUR-LEX, 2018) regarding the strategic goal of EU to a complete elimination of the disposal of municipal wastes one of the objectives of the present research is to reuse the slag waste dumped in landfills.

The aim of the study was to improve the pH of acidic soil (5.2) using a mixture formed by slag waste dumped in landfill and of granulated blast furnace slag. The novelty of our research consists in the usage of a part of the slag dumped in landfill without any selective separation by source of slag which is a costly process but also, solving an important environment issue regarding decommissioning of slag dump in amelioration of soil acidity.

## Results and discussion

### Structural characterization of slag samples

**Figure 1:**
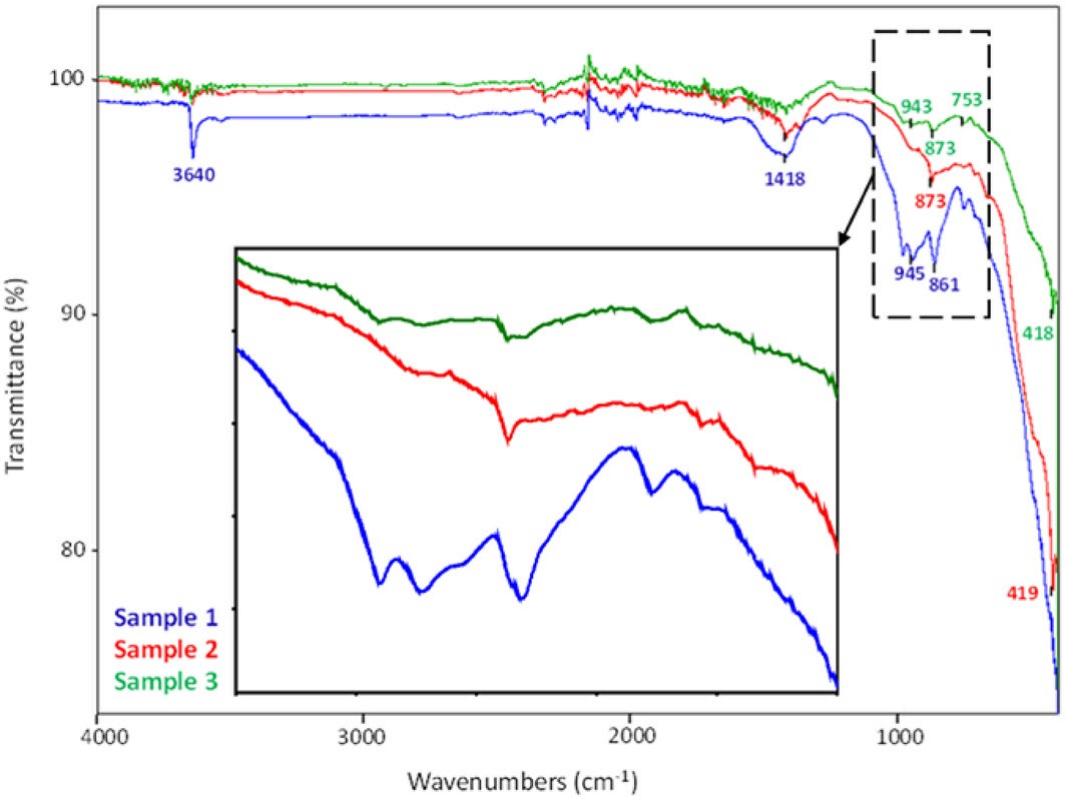
FTIR spectra of slag samples.

The FTIR spectra of granulated blast furnace slag (Sample 1), waste slag dumped in landfill (Sample 2) and combination of both 50% granulated blast furnace slag + 50% waste slag dumped in landfill (Sample 3) are presented in Fig. 1.

By analysing the spectrum (detailed figure) in the range of 700–1100 cm^−1^, it can be found that there are obvious absorption peaks in the spectrum of all the slag samples. The granulated blast furnace slag shows the characteristic absorption bands at 3640, 1418, 980, 944, 861, 753 and 710 cm^−1^. The band at 3640 cm^−1^ is assigned to the stretching vibration of the hydroxyl group originated from the weakly absorbed water molecules on the slag surface^24^. The characteristic absorption bands at 1418, 861 and 710 cm^−1^ are ascribed to the asymmetric stretching mode and bending mode of carbonate group, respectively and the band at 980 cm^−1^ are attributable to the stretching vibrations of Si–O^25^. The band at 944 and 752 cm^−1^ represent the internal vibration of [SiO_4_]^4−^ and [AlO_4_]^5−^ tetrahedral and comes from Si (Al)–O-antisymmetric stretching vibration^26^.

The different vibration modes for the sample of waste slag can be observed in the FTIR spectrum. The absorption bands shown are at 1418, 873, 712, 667 and 419 cm^−1^. The peak at 1418 cm^−1^ is assigned to the asymmetric stretching mode and bending mode of carbonate group. Calcite phase is confirmed by characteristic peaks at 712 cm^−1^ (ʋ_2_ out of plane bending vibration of the CO_3_^−2^ ion) and 873 cm^−1^ peak (ʋ_2_ split in-plane bending vibrations of the CO_3_^−2^ ion^27^. Calcium aluminate phase is identified by characteristic peak at 419 cm^−1^^28^. Peak around 667 cm^−1^ is described as absorption band for different M–O (metal oxide) such as Al–O, Fe–O, Mg–O etc.^29^.

In the case of combination of both 50% granulated blast furnace slag and 50% waste slag dumped in landfill the intensity of absorption peaks is smaller in comparison with Sample 1 and Sample 2 of slag. The characteristic absorption peaks (978 and 753 cm^−1^) which correspond with characteristic peaks of Sample 1 are shifted compared to the Sample 1, assigned to the stretching vibrations of Si–O and to the Si (Al)–O-antisymmetric stretching vibration, respectively, can provide important evidence of chemical interaction between Sample 1 and Sample 2. The decrease of the intensity of the bands appearing at 875 and 709 cm^−1^ cans be attributed to overlapping the vibrations of the CO_3_^−2^ ion from calcite phase.

**Figure 2:**
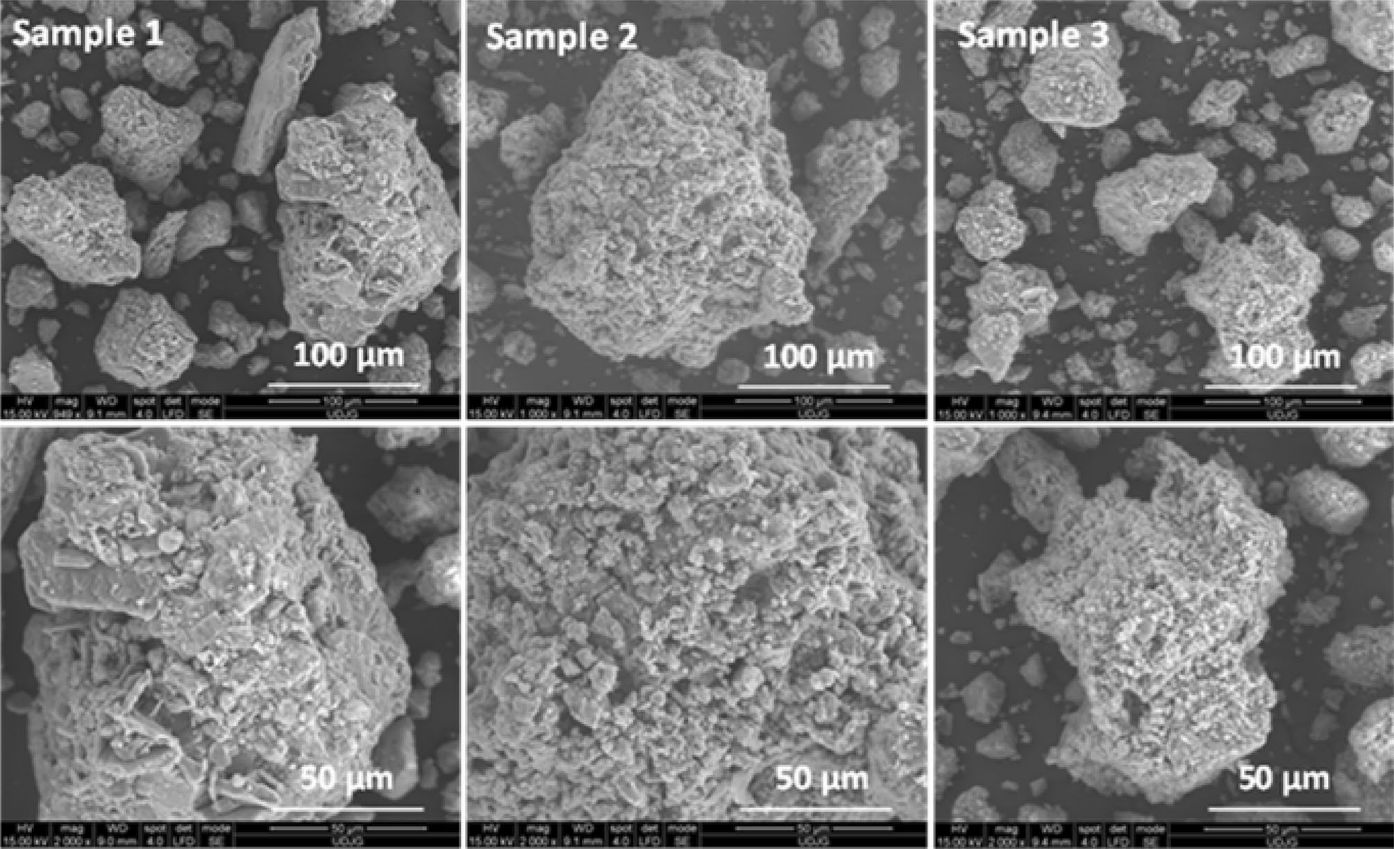
SEM images of slag samples.

Figure 2 presents the SEM micrographs of the slag samples (Sample 1–3). One can see the characteristic morphology- the sizes and the forms of the slag samples.

At larger magnifications it can be observed that the surface is rough and uneven, and one can notice rounded grain-like rugged formations. The slag samples display aggregated particles with average diameter of a few microns. Also, in these rounded formations it can be seen different morphologies like spheres, rods, boards specific each compound/phase from metallurgical slags.

**Figure 3:**
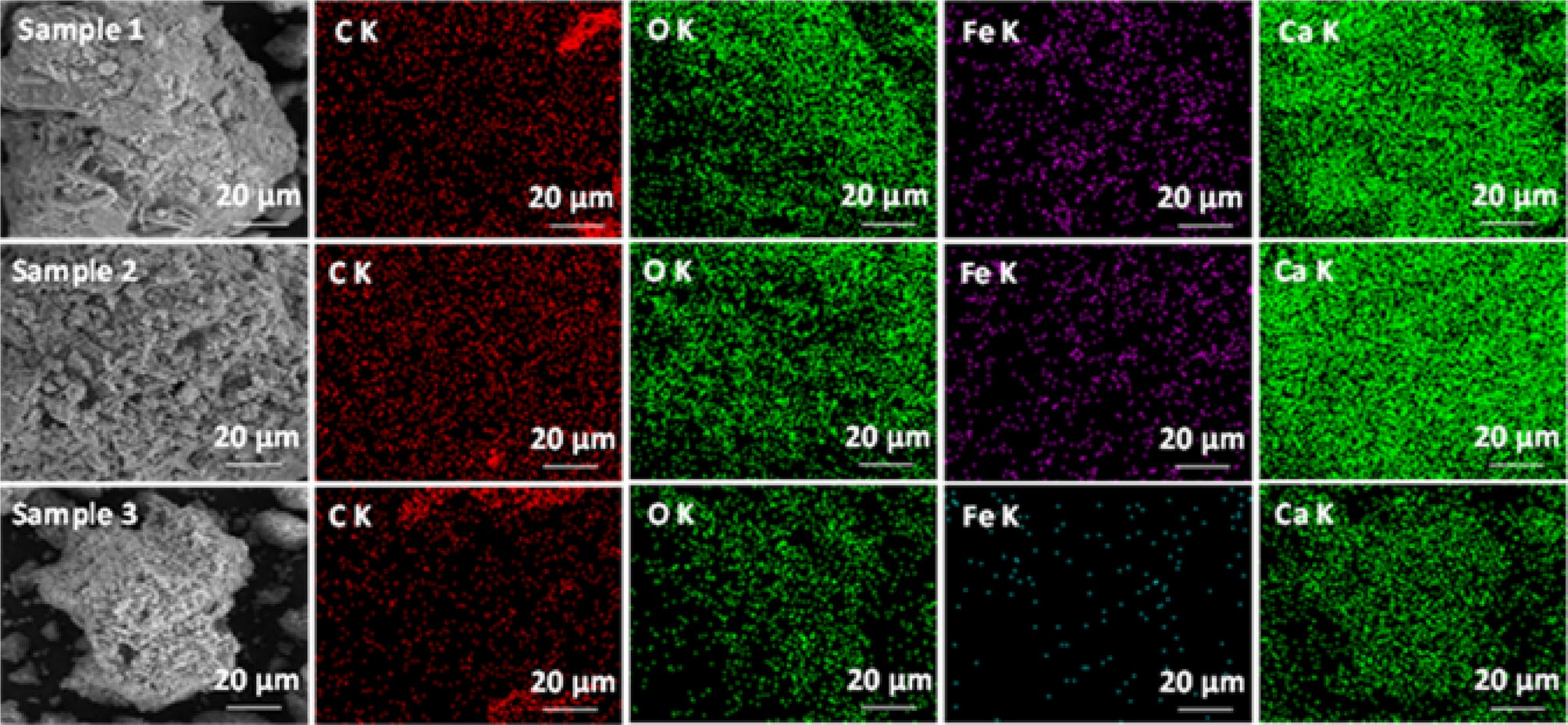
EDX elemental map of slag samples.

Figure 3 illustrates the EDX elemental analysis of granulated blast furnace slag (Sample 1), waste slag dumped in landfill (Sample 2) and combination of both 50% granulated blast furnace slag + 50% waste slag dumped in landfill (Sample 3).

One can observe that the predominant elements in the examined area are constated in carbon, oxygen, calcium, and iron, confirming the FTIR spectra.

**Figure 4:**
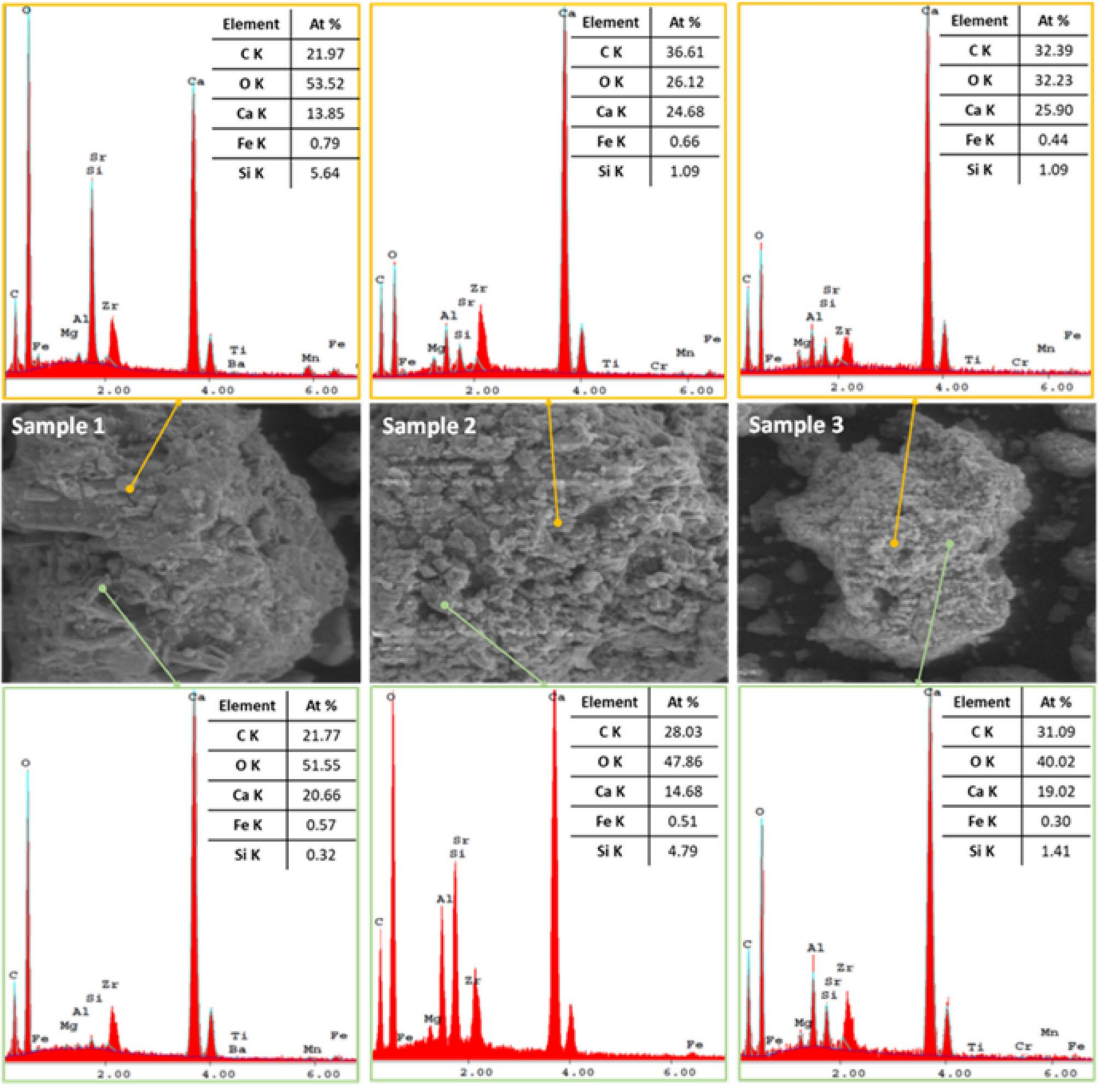
EDX spectra analysis of slag samples.

Figure 4 shows EDX spectra of slag samples recorded on different selected punctual area, to obtain more information about the elemental composition of specific areas. For all the tested slag samples have similar elements content.

The selected punctual areas are highlighted thus: the spheric structure are with yellow line and the structure like boards are with green line for all the analysed slag samples. In the case of Sample 1 for both structures the values of chemical elements present are similar and the silicon has a higher value at spheric structure which can be correlated with the presence of silica (SiO_2_). The higher content of calcium reveals that the Sample 1 is blast furnace slag dominated by calcium and silicon compositions. In the case of slag dumped in landfill (Sample 2) the content of carbon increase for both structures and some chemical elements like titanium, barium, manganese doesn`t appear in EDX spectra and the explanation for this phenomenon is that the slag was dumped in landfill for more than 30 years. One can observe for combination of both 50% granulated blast furnace slag + 50% waste slag dumped in landfill (Sample 3) that the values of all the chemical elements for both spheric and board-like structure are between the first two samples, confirming the FTIR spectra regarding chemical interaction between Sample 1.

**Figure 5:**
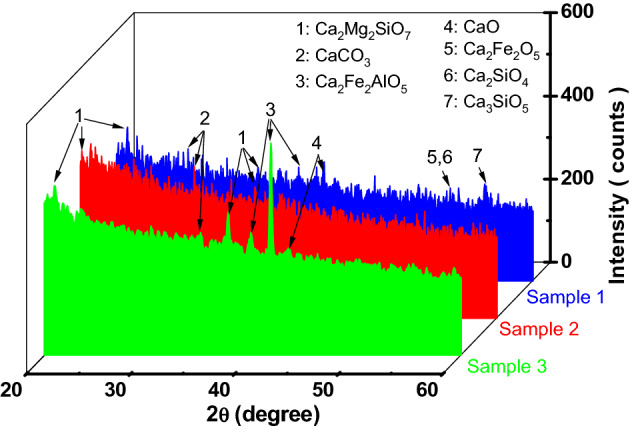
X-ray diffraction patterns of slag samples.

XRD patterns of the slag samples with the phases identified are shown in Fig. 5. Sample 1 show minor peaks of free CaO and MgO, which may be deleterious and cause reduction in strength. The phases and amorphous contents of the Sample 1 granulated blast furnace slag are broadly consistent with literature^30^. Sample 3 of slag consists of crystalline phase – Ca_2_Mg_2_SiO_7_, Ca_2_Fe_2_AlO_5_, CaCO_3_ and CaO as observed by the XRD analysis. In terms of the relations of phase thermal equilibrium, the compounds identified form an isomorphic series of melilites that is specific to basic metallurgical slags.

In Table 1 are presented the values expressed as ppm of chemical element detected in slag samples (Sample 1, 2 and 3).

**Table 1:** XRF analysis of the slag samples.

Element detected	Sample 1(slag)		Sample 2 (slag)		Sample 3 (slag)	
	ppm	+ /−	ppm	+ /−	ppm	+ /−
Mg/MgO	1500/2487	162	5800/9619	629	8500/14,097	922
Al/Al_2_O_3_	1400/2644	151	16,100/30,416	1747	16,600/31,361	1801
Si/SiO_2_	3200/6844	347	9100/19,463	987	10,700/22,885	1161
Mn/MnO	6100/7877	661	4600/5942	499	4400/5683	477
Fe/Fe_2_O_3_	11,400/16,298	1237	10,900/15,583	1182	18,900/27,021	2050
Ca/CaO	298,300/417,410	32,369	295,800/413,912	32,098	364,300/509,763	39,531

The results show a large quantity of calcium in all three samples of slag. Also, the elements detected such as Fe, Al, Mg and Si are in accordance with XRD spectra.

### Physical–chemical characterization of soil-slag mixtures

The chemical composition of the major elements that compound the soil, soil- slag and slag samples was determined by XRF. The values expressed as ppm of chemical elements are presented in Table 2. In the case of soil sample the content of the main constituents is iron, titanium, manganese, and potentially toxic elements (PTE) such as arsenic, zinc, copper, and cobalt. For soil-slag 1 with weight ratio soil: slag (1:1) it can be observed the disappearance of the potentially toxic elements (PTE) founded in soil sample and the decrease of concentration value of zinc. When the weight ratio of slag increases at 3 (soil-slag 2 sample) the values of main component increased in accordance with values of slag sample, but in the case of soil-slag 3 sample where the weight ratio of soil is bigger (3) it can be observed the cobalt presence. Based on these XRF results we can say that take place an elimination of potentially toxic elements in contaminated soil by applying slag in a bigger proportion.

**Table 2:** XRF analysis of the soil-slag samples.

Element detected	Samples									
	Soil		Soil-slag 1		Soil-slag 2		Soil-slag 3		Slag (sample 3)	
	ppm	+ /−	ppm	+ /−	ppm	+ /−	ppm	+ /−	ppm	+ /−
Fe	30,505	449	60,148	982	64,656	1095	43,099	660	86,862	1583
Ti	4961	397	2961	466	1978	482	3508	419	–	–
Mn	585	45	11,469	250	14,608	312	6698	158	19,250	424
Cu	28	9	–	–	–	–	–	–	–	–
Zn	101	7	89	8	108	9	100	8	–	–
Zr	327	6	189	5	167	5	241	6	428	10
As	10	3	–	–	–	–	–	–	–	–
Co	494	96	–	–	–	–	358	119	–	–
Others	263	11	217	11	230	20	226	11	160	6

With the aid of a pH meter, CONSORT C 533 the important parameters of soil and slag solutions were measured as: the pH, conductivity, and the salinity, as shown in Table 3. The data presented in Table 3 suggest that the soil sampled has the pH = 5.2 corresponding to a medium acid soil, which does not sustain a high fertility and is not able to offer proper conditions for crops. Also, the pH of soil has important influence on soil fertility, decreases the availability of essential elements and the activity of soil microorganisms which can determine calcium and magnesium deficiency in plants and decreases phosphorous availability. The pH value of slag solution (12.5) corresponds as strongly basic character which is beneficial in amelioration process of acidic soils and the presence of this type of slag sustain the improving of soil characteristics, too. For the soil-slag samples the pH value increase with the increasing of the weight ratio of slag and the mixtures soil-slag obtained can be framed into the category of weakly alkaline soils.

**Table 3:** The physical–chemical characteristics of soil and slag solutions.

Sample	Parameters		
	pH	Conductivity[mS/cm]	Salinity [ppt]
Soil solution	5.2	0.29	0.2
Slag solution	12.5	6.38	3.6
Soil-slag 1	8.02	2.55	1.3
Soil-slag 2	8.53	5.65	2.1
Soil-slag 3	7.78	0.82	0.4

The data given in Table 4 show that the humidity of soil is bigger and decreases in soil-slag samples with adding of slag content. The values of total soil-slag porosity are between 40 and 50% and depends on the density and apparent density of the soil being influenced by the mineralogical composition, the content of organic matter and the degree of compaction and loosening of the soil, the crystalline structure of soil minerals.

**Table 4:** The physical–chemical characteristics of soil-slag samples.

Sample	Parameters	
	Humidity [%]	Total porosity [%]
Soil	12.07	50
Soil-slag 1	5.59	40
Soil-slag 2	3.00	42
Soil-slag 3	8.42	46

Considering the structural and morphological characterization of the investigated slag samples we propose a recipe of blast furnace slag and of waste slag dumped in landfill in accordance with the waste directive 2008/98/EC regarding the strategic goal of EU to a complete elimination of the disposal of wastes. The slag dump of Steel Plant of Galati has an enormous quantity of unused waste slag which may be mixed with granulated blast furnace slag, to save the natural resources used as raw materials in the metallurgical technological process.

The presence of Ca^2+^ in the composition of the slag can maintain high alkalinity in the soil for a long time in the natural environment. The alkaline pH of the soil may contribute to a decrease the available concentration of heavy metals by reducing metal mobility and bonding metals into more stable fractions. One of the objectives of this research is improving the quality of the environment by using the mixture between two different slags on agricultural lands and reintroducing them in the agricultural centre, especially in acid soils. Acidic soils are characterized by an acidic pH that has spread in recent years due to excessive fertilizers or far too aggressive work^31^. The production is significantly influenced, and the treatment of acid soils is usually done using a series of natural materials (lime, dolomite), the consumption being approx. 20 t/hectare depending on the acidity of the soil and the nature of the plants grown on the respective surfaces.

Our research consists in improving the characteristics and qualities of the acidic soils and helping to reintroduce it into the agricultural circuit by transforming a waste into a new material friendly-environmental, the mixture of blast furnace slag and waste slag dumped in landfill.

## Materials and methods

### Material and sample preparation

#### Slag sample preparation

In this research, the slags were collected from the largest Integrated Steel Company of Galati. The Galati Steel Plant produces a wide range of quality flat products (plates, coils, galvanized sheets & coils, and organic coated products) as well as welded tubular products and supplies, for customers across Romania, the Balkans and elsewhere in Europe.

The types of slag used for characterization were granulated blast furnace slag (Sample 1) and waste slag dumped in landfill (Sample 2). The slags were coarse in size, and it were crushed and milled in different size fractions in the range 1.25 mm–71 μm.

The granulated blast furnace slag is obtained by water quenching method when at the high temperature range 1250–1650 °C of blast furnace slag is conventionally treated in cold water by forming a non-crystalline granular material that differs from the source to source in function of the quality of the raw material used and the method of production. The waste slag dumped at the Galati Steel Plant landfill that was used for this study represents a significant source of pollution because it is not recycled. Following the results obtained of the samples enumerated above, we saw the benefits regarding of each sample and we made a new recipe of a combination of both 50% granulated blast furnace slag + 50% waste slag dumped in landfill (Sample 3) for the obtaining new trends and to recycle the slag dumped in enormous quantities. The granulated blast furnace slag taken immediately after cooling in water, with water addition was mixed with waste slag dumped in landfill, which previously was crushed with a mortar grinder to obtain Sample 3. The combination of obtained slag (Sample 3) was used in soil-slag mixture for correction of soil acidity.

#### Soil-slag mixture preparation

**Figure 6:**
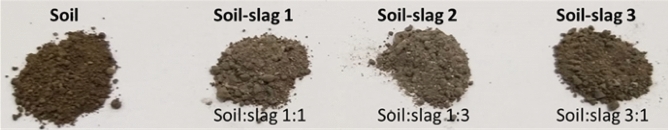
Images of mixtures of soil-slag samples.

The topsoil (0–0.25 m) was sampled from a commune of Galati County of Romania, (no specific permissions were required for soil sampling in this location and the field in this study did not involve endangered or protected species). The collected soil was crushed and milled in different size fractions. The soil and the new recipe of the combination of the two types of slags (Sample 3), mentioned above, were combined in different proportions, to prepare 3 mixtures, illustrated in Fig. 1, with weight ratio soil: slag such as 1:1 (Soil -slag 1), 1: 3 (Soil-slag 2) and 3: 1 (Soil-slag 3). The photo images of soil-slag samples are presented in Fig. 6.

### Characterization techniques

FTIR spectroscopy is a well-established technique for elucidating the structure of glassy and crystalline aluminosilicates (aluminosilicate glasses and zeolites respectively). Fourier transform infrared (FTIR) spectra of the slag samples were recorded using a NICOLET IS50 FT-IR SPECTROMETER (THERMO SCIENTIFIC) equipped with a built-in ATR accessory, DTGS detector and KBr beam splitter. The scan range was set of 4000–400 cm^−1^ with a resolution of 4 cm^−1^ and the number of scans was 32 times. The reference taken for the background spectrum before each sample was air and the ATR plate was cleaned with ethanol solution after each spectrum. To verify that no residue from the previous sample remained, a background spectrum was collected each time and compared to the previous background spectrum. The FT-IR spectrometer was placed in a temperature-controlled (21 °C) air-conditioned chamber.

The morphology and elemental composition of the investigated slag were examined by scanning electron microscopy coupled with energy dispersive X-Ray (SEM/EDX) spectroscopy using a FEI Q 200 MICROSCOPE IN LOW VACUUM. Before examination, the samples were coated with 4 nm thick conducting layer of Au using a SPI-Module sputter coater system.

X-ray diffraction spectra (XRD) were recorded using the DRON 3 EQUIPMENT (BOUREVESTNIK INC., ST. PETERSBURG, RUSSIA) and a Cobalt anode (Co, λKa = 1.790300 Å, with setting of: voltage (U) of 30 kV and an intensity (I) of 20 mA, with a step of 0.05 o/s and a range between 20 and 65-degree time exposure of 3 s, and total time/sample 1 h and 13 min.

The soil-slag samples were characterized by X-Ray Fluorescence (XRF) (INNOV-X-SYSTEMS ALPHA-4000) for a quantitative determination of major and trace element concentrations in soil material using a calibration with matrix-matched standards.

The pH of the soil and slag samples was determined with a multi-parameter analyser CONSORT C 533. The pH was measured in solution with weight ratio soil / slag: distilled water 1: 5, decantation for 24 h and filtration.

The humidity, U, of the soil-slag samples was calculated by Eq. (1) according with Standard SR 648 2002.

1 $$ U = \frac{{m - m_{1} }}{m} \times 100\user2{ }\left[ \user2{\% } \right] $$

where m is the mass of the sample taken in determination, in grams, m1 is mass of sample remaining after drying for two hours at 105 °C, in grams.

The porosity of soil is the volume of space in between the mineral particles of soil. The total porosity, PT, of the soil-slag samples was calculated by Eq. (2).

2 $$ PT = \frac{D - DA}{D} \times 100 \left[ \% \right] $$

where D is the density and DA is the apparent density of the soil-slag samples taken in determination in a graded cylinder.

## Conclusions

Soil pH is a very important parameter because it plays an important role in the mobility of heavy metals, and an acidic pH is accompanied by dissolution processes of minerals and hydrated Fe, Mn and Al oxides on which heavy metal ions are adsorbed. The pH and its changes undoubtedly play a significant role of the soil environment because the heavy metals may become easily available to plants. The agricultural land resources are an issue worldwide and through this investigative study we showed that the mixture of blast furnace slag and waste slag dumped in landfill can help remediation of the soil acidity. The obtained results of the new slag mixture support the improvement of acid soils with positive effects on the characteristics and qualities of the soil. By using a precent of the slag waste combining with granulated slag can solve two current issues, one by disposal of landfilled slag wastes and the second one is to make them useful in the acid soils amelioration process. If we consider the cost–benefit analysis of the future environment in terms of landfills, the quality of human health of the population is more substantial that is not measured financially.

The results from this study together with large scale environmental issues poses significant long for further studies which consist in eco-toxicity tests of slag-soils mixtures on the adsorption and immobilization of potential toxic elements from soil for sustainable agriculture.

## Supplementary Information

Supplementary Information.

## Data Availability

All data analysed during this study are included in this published article and its supplementary information files.
