# Supplementary material for: Waste slag benefits for correction of soil acidity
Source: Sci Rep. 2022 Sep 26;12:16042. doi: 10.1038/s41598-022-20528-6 (PMC9512849; doi:10.1038/s41598-022-20528-6)
Supplement: Supplementary file 1 — Supplementary Information. [file 41598_2022_20528_MOESM1_ESM.docx]

Waste Slag Benefits for Correction of Soil Acidity

Viorica Ghisman^1^, Alina Crina Muresan^1^, Daniela Laura Buruiana^1^, Elena Roxana Axente^2^

^1^ Interdisciplinary Research Centre in the Field of Eco-Nano Technology and Advance mate-rials CC-ITI, Faculty of Engineering, “Dunarea de Jos” University of Galati, 47 Domneasca, 800008 Galati, Romania

^2^ Medicine and Pharmacy Faculty, “Dunarea de Jos” University of Galati, 47 Domneasca, 800008 Galati, Romania *Correspondence to*: Daniela L. Buruiana ([Daniela.buruiana@ugal.ro](mailto:Daniela.buruiana@ugal.ro))

The FTIR spectra of the raw data of granulated blast furnace slag (Sample 1), waste slag dumped in landfill (Sample 2) and combination of both 50% granulated blast furnace slag + 50% waste slag dumped in landfill (Sample 3) are presented in Fig. S1.

**Figure S1:** FTIR spectra of slag samples.

In the tables below (Table S1-S3) are presented the raw data obtained for slag samples with FT-IR SPECTROMETER.

**Table S1:** FT-IR peaks for Sample 1 (granulated blast furnace slag)

| Position | Intensity |
| --- | --- |
| 861.98 | 92.213 |
| 945.04 | 92.375 |
| 1418.21 | 96.747 |
| 3640.87 | 96.710 |
|  |  |

**Table S2:** FT-IR peaks for Sample 2 (waste slag dumped in landfill)

| FIND PEAKS:  Spectrum: **SAMPLE (PROBA) 2**  Region: 4000.00 400.00  Absolute threshold: 98.560  Sensitivity: 50  Peak list: | |
| --- | --- |
| Position | Intensity |
| 419.04 | 78.314 |
| 873.18 | 95.662 |
| 1418.32 | 97.565 |

**Table S3:** FT-IR peaks for Sample 3 (mixture of 50% granulated blast furnace slag + 50% waste slag dumped in landfill)

| FIND PEAKS:  Spectrum: **SAMPLE (PROBA) 3**  Region: 4000.00 400.00  Absolute threshold: 98.462  Sensitivity: 70  Peak list: | |
| --- | --- |
| Position | Intensity |
| 402.96 | 90.310 |
| 418.79 | 90.054 |
| 432.12 | 91.421 |
| 443.91 | 91.668 |
| 469.29 | 92.770 |
| 753.12 | 98.221 |
| 873.20 | 97.843 |
| 943.54 | 98.131 |

In figures S2-S4 shows EDX spectra of slag samples recorded over the entire surface of the analysed area.

**Figure S2:** EDX spectra analysis of slag sample 1.

**Figure S3:** EDX spectra analysis of slag sample 2.

**Figure S4:** EDX spectra analysis of slag sample 3.

In figures below are showed XRD patterns of the raw data of granulated blast furnace slag (Sample 1-Figure S5), waste slag dumped in landfill (Sample 2--Figure S6) and combination of both 50% granulated blast furnace slag + 50% waste slag dumped in landfill (Sample 3--Figure S7).

**Figure S5:** XRD spectra analysis of slag sample 1

**Figure S6:** XRD spectra analysis of slag sample 2

**Figure S7:** XRD spectra analysis of slag sample 3
